# Neighborhood sampling: how many streets must an auditor walk?

**DOI:** 10.1186/1479-5868-7-20

**Published:** 2010-03-12

**Authors:** Tracy E McMillan, Catherine Cubbin, Barbara Parmenter, Ashley V Medina, Rebecca E Lee

**Affiliations:** 1 PPH Partners, 7710 W Sweetwater Tr, Flagstaff, AZ 86001, USA; 2 School of Social Work, Population Research Center, University of Texas at Austin, 1 University Station G1800, Austin, TX 78712, USA; 3 Department of Urban and Environmental Policy and Planning, Tufts University, 97 Talbot Avenue, Medford, MA 02155, USA; 4 Texas Obesity Research Center, Department of Health and Human Performance, University of Houston, 3855 Holman St, Garrison Gymnasium Rm 104, Houston, TX 77004, USA

## Abstract

This study tested the representativeness of four street segment sampling protocols using the Pedestrian Environment Data Scan (PEDS) in eleven neighborhoods surrounding public housing developments in Houston, TX. The following four street segment sampling protocols were used (1) all segments, both residential and arterial, contained within the 400 meter radius buffer from the center point of the housing development (the core) were compared with all segments contained between the 400 meter radius buffer and the 800 meter radius buffer (the ring); all residential segments in the core were compared with (2) 75% (3) 50% and (4) 25% samples of randomly selected residential street segments in the core. Analyses were conducted on five key variables: sidewalk presence; ratings of attractiveness and safety for walking; connectivity; and number of traffic lanes. Some differences were found when comparing all street segments, both residential and arterial, in the core to the ring. Findings suggested that sampling 25% of residential street segments within the 400 m radius of a residence sufficiently represents the pedestrian built environment. Conclusions support more cost effective environmental data collection for physical activity research.

## Findings

Neighborhood context has been associated with health and physical activity (PA) [1-13]. Studies of specific neighborhood characteristics, including pedestrian pathways, reduced automobile traffic, and aesthetic appeal, and their association with PA [14], have yielded rigorous instrument development, validation and implementation to increase understanding of the role the built environment plays in PA [15]. Nevertheless, there remain unresolved issues concerning specific sampling and data collection protocols that have implications for future research, promotion and policy.

Although some municipalities collect and compile GIS data about the built environment that aid PA research, most do not. Very few have detailed data such as sidewalk condition or pathway obstructions, and the quality and consistency of the GIS data vary widely [16]. Many environmental audit instruments have been developed to address these limitations. Four frequently used instruments include the Systematic Pedestrian and Cycling Environmental Scan (SPACES) [17]; the Irvine Minnesota Inventory (I-M) [18]; the Analytic Audit Tool and Checklist Audit Tool (SLU) [19]; and the Pedestrian Environment Data Scan (PEDS) [20]. Each has adequate reliability and provides a rich assortment of micro-scale environment data. The principal limitation to these instruments is the time and cost involved in data collection. PEDS has the lowest data collection time of these audit instruments [20], averaging 3-5 minutes per segment, compared to 10 to 20 minutes for the I-M and SLU. A full inventory of street segments within a quarter mile radius of a selected address can exceed 100 segments -- 17-34 hours for data collection per neighborhood.

Environmental audits on the complete census of streets in a neighborhood may be unnecessary, as there is likely substantial homogeneity within street types in a neighborhood, particularly residential streets. Pikora and colleagues noted that the lack of variation of built environment characteristics among assessed segments "resulted in skewed distributions of responses to some items," which led to a high level of chance agreement and low kappa scores for these items [17]. To address this, Agrawal, Schlossberg, and Irvin suggest that data collection be both streamlined and customized within street types [21]. For example, characteristics such as sidewalk, traffic volumes, posted speeds, number of lanes of traffic and safe crossings will vary more significantly across a sample of arterial streets than of residential streets. Therefore, although a full sample of arterials may be necessary to capture this variability, a reduced sample of residential street segments may be possible due to less variation from one street segment to the next.

This study tested four different street segment sampling protocols using a gently modified version of the PEDS to determine whether abbreviated data collection protocols can sufficiently and accurately represent the pedestrian built environment. Analyses were conducted to determine whether (1) the immediately proximal neighborhood (within 400 meters of the residence) is notably different from nearby neighborhoods (by adding an additional 400 meters to the radius) that might impact daily physical activity and (2) it is necessary to sample the complete census of all residential street segments within the 400 meter radius buffer. Our hypothesis in both cases was that no variation in the tested characteristics would exist, thereby supporting the scientific integrity of sampling strategies using abbreviated data collection.

Eleven neighborhoods in the City of Houston were selected for this study. Neighborhoods were defined as the area within an 800 meter radius circumscribed around a public housing development managed by the Houston Housing Authority in Houston, Texas [Eugeni, Baxter, Lee: Disconnections of African American public housing residents: Connections to Physical Activity, Dietary Habits and Obesity, submitted; [22]]. Housing developments are affordable rental housing for families, seniors, and persons with disabilities, federally-funded and managed by the Houston Housing Authority. Defining the neighborhood as the area within the boundaries of the circle has several advantages [23,24]. First, it captures all areas to which a resident may be exposed on a daily basis during both foot and automobile travels. Second, the straight line distance allows for capture of distance traveled on footpaths and other "short cut" routes that may not be captured by using a street network or aerial satellite photography strategy. Third, it may reduce the effect of spatial correlation that arises from using census boundaries where points near the boundary of the census area are influenced by factors in adjacent census areas, as housing developments were selected to be at least 1600 meters apart. All housing development neighborhoods were located in urban areas that were predominantly lower income, with higher proportions of ethnic minorities.

The neighborhoods varied by socioeconomic status (SES) index, racial ethnic concentration and street node density. To characterize neighborhood-level SES, we constructed an index based on one of the author's (CC) previous work [25]. Five variables from U.S. Census block-group level data from the year 2000 for Harris County were standardized and then summed with equal weights to compute the index: percentage aged 25 and older with less than a high school education, median annual family income, percentage blue collar workers, percentage unemployed, and median housing value. Correlations among the five variables ranged from 0.35 to 0.89 and principal components analysis revealed that the five variables explained 69% of the total variance. Harris County and HD neighborhood socio-demographic characteristics are presented in Additional File 1: Table S1 [Harris County and HD Neighborhood socio-demographic characteristics]. All street segments in each neighborhood were identified using ArcVIEW. Prior to going into the field, neighborhoods were mapped using GIS technology. Street segments were numbered in preparation for field assessment and assessed using the PEDS [20]. This instrument assesses a number of street characteristics associated with physical activity, in particular, pedestrian activities and bicycling. Trained field assessors were deployed to neighborhoods in teams of two following established safety protocols [24]. Each segment was carefully assessed and rated using the operational definitions from the PEDS instrument. Since a full sample of street segments was initially collected for this project, street segments were not identified as residential or arterial prior to conducting data collection. The road type of the street segment was recorded at the time of the assessment. Residential streets were defined as moderate to low volume roads that carry less than 5,000 cars per 24 hour period. Arterial streets were defined as high volume, main roads that carry approximately 5,000-10,000 cars per 24 hour period. On and off ramps were not included in the sample of residential street segments. However, it is important to note that although the functional classification of roadways (e.g. arterial vs. residential) may be available in GIS datasets obtained from municipalities, this should be verified in the field prior to sampling procedures and before completing environmental audits.

Analyses were conducted on five key variables associated with walking: sidewalk presence; observer ratings of attractiveness and safety for walking; connectivity; and number of traffic lanes (proxy for speed/volume). HD neighborhood pedestrian built environment characteristics are described in Additional File 2: Table S2 [HD Neighborhood pedestrian built environment characteristics]. The rating of attractiveness pertained to finding the area aesthetically pleasing and to the existence of destinations. It answered the question: "would you want to walk/bike this segment?" The rating of safety for walking took into consideration not only walking along the sidewalk but crossing the street. "Would a child be safe walking the segment?" Response to safety for cycling considered road attributes such as speed limits and presence of bicycle facilities. All data were collected, entered into an Access database and proofed by two research team members using established protocols [22,24].

**Figure 1:**
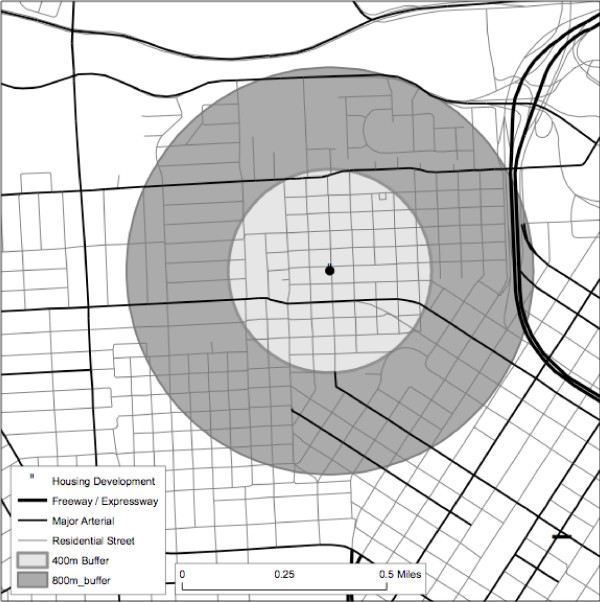
**Map of Housing Development Neighborhood**. Figure 1 graphically displays the geographic comparisons between all segments contained within the 400 meter radius buffer from the center point of the housing development (the core) with the segments contained between the 400 meter radius buffer and the 800 meter radius buffer (the ring).

Chi square analyses were used to compare similarity in the five key variables for different geographic areas and different sampling strategies. First, we compared all segments contained within the 400 meter radius buffer from the center point of the housing development (the core) with the segments contained between the 400 meter radius buffer and the 800 meter radius buffer (the ring) as illustrated in Figure 1. Second, we compared all residential segments in the core with three different percentages of randomly selected residential street segments in the core: 75%, 50% and 25%--to determine whether a sample of segments fewer than 100% would still be representative of the pedestrian environment. Analyses were conducted for all neighborhoods combined and separately. Figure 1 graphically displays the geographic comparisons between all roads within the 400 m and 800 m buffers.

As presented in Additional File 3: Table S3 [All HD and HD Neighborhood core vs. ring comparison of pedestrian built environment characteristics], a few significant differences were seen between the core and the ring. There was significant variability in sidewalk presence in two neighborhoods, connectivity varied in four neighborhoods, and traffic lanes varied in three. Attractiveness for walking varied significantly between core and ring in three neighborhoods and feelings of walking safety significantly varied in four neighborhoods. General patterns indicated that ratings of attractiveness and safety were lower in the core than in the ring.

There were no significant differences in the comparison of the residential street segments in the core compared to a random sample of residential segments (75%, 50% and 25%) as presented in Additional File 4: Table S4 [Comparisons of core vs. random sample of core residential segments (75%, 50% and 25%)].

The goal of this study was to determine the extent of sampling necessary to provide a representative sample of the built environment in residential neighborhoods. Some differences were found when comparing the core to the ring, likely due to the increased variability of arterials in this broader street sample covering a larger geographic area. No differences existed when residential segment samples of 75%, 50% and 25% were compared to the census of all residential streets in the core. These findings suggest that sampling as few as 25% of residential street segments within the 400 m radius of a residence may sufficiently represent the pedestrian environment and provides support for abbreviated data collection schemes of homogeneous street data for efficiency and cost-savings.

Although this study only included 11 neighborhoods, the systematic protocols and considerable detail provided comprehensive data with good variability. Strengths included detailed data collection, trained data collectors, and a complete census of street segments. A limitation of this study is that findings may not be generalizable to other Houston areas not surrounding a housing development or to older cities that have greater historical diversity in micro neighborhood design; thus, this study should be replicated.

Findings suggest that future studies may reduce the burden of exhaustive neighborhood data collection as a relatively small sample of the neighborhood residential street segments may appropriately represent the residential built environment. Arterial streets and streets with more mixed use introduce much greater variability and richness in datasets, and future studies are needed to capture the depth and influence of arterial streets in the pedestrian environment.

## Competing interests

The authors declare that they have no competing interests.

## Authors' contributions

REL led the study and assisted with writing. TM assisted with the study and writing. CC completed analyses and assisted with the study and writing. BP led the cartography and assisted with the analyses and writing. AM assisted with the study and writing. All authors read and approved the final manuscript.

## Supplementary Material

Additional file 1 ***Table S1*. Harris County and HD Neighborhood socio-demographic characteristics**. Table S1 describes the neighborhood socio-demographic characteristics of Harris county and of each housing development neighborhood. Click here for file

Additional file 2 ***Table S2*. HD Neighborhood pedestrian built environment characteristics**. Table S2 describes the pedestrian built environment characteristics of each housing development neighborhood. Click here for file

Additional file 3 ***Table S3*. All HD and HD Neighborhood core vs. ring comparison of pedestrian built environment characteristics**. Table S3 describes the core vs. ring comparisons of pedestrian built environment characteristics of all housing development neighborhoods and of each housing development neighborhood. Click here for file

Additional file 4 ***Table S4*. Comparisons of core vs. random sample of core residential segments (75%, 50% and 25%)**. Table S4 describes the comparisons of residential street segments in the core and a random sample of 75%, 50%, 25% residential segments in the core, respectively. Click here for file
