# Supplementary material for: Neighborhood sampling: how many streets must an auditor walk?
Source: Int J Behav Nutr Phys Act. 2010 Mar 12;7:20. doi: 10.1186/1479-5868-7-20 (PMC3224902; doi:10.1186/1479-5868-7-20)
Supplement: Additional file 1 — Table S1. Harris County and HD Neighborhood socio-demographic characteristics. Table S1 describes the neighborhood socio-demographic characteristics of Harris county and of each housing development neighborhood. [file 1479-5868-7-20-S1.DOC]

| *Table 1*. Harris County and HD Neighborhood sociodemographic characteristics | | | | | | | | | | | | |
| --- | --- | --- | --- | --- | --- | --- | --- | --- | --- | --- | --- | --- |
|  | **Harris County** | **HD1** | **HD2** | **HD3** | **HD4** | **HD5** | **HD6** | **HD7** | **HD8** | **HD9** | **HD10** | **HD11** |
| **Characteristic** |  |  |  |  |  |  |  |  |  |  |  |  |
| % blue collar | 37 | 66 | 53 | 51 | 46 | 59 | 46 | 59 | 67 | 28 | 51 | 63 |
| % < HS education | 25 | 61 | 48 | 39 | 32 | 39 | 29 | 47 | 61 | 24 | 53 | 62 |
| Median family income ($) | 49,004 | 26,323 | 72,565 | 22,864 | 42,798 | 26,395 | 36,016 | 35,078 | 30,719 | 55,709 | 29,254 | 28,632 |
| Median housing value ($) | 87,000 | 37,727 | 20,240 | 57,236 | 118,650 | 39,950 | 89,429 | 64,282 | 50,825 | 148,475 | 23,157 | 42,875 |
| % unemployed | 6 | 14 | 22 | 18 | 11 | 17 | 6 | 9 | 13 | 10 | 13 | 11 |
| % Black | 18 | 16 | 36 | 92 | 20 | 94 | 19 | 18 | 11 | 57 | 61 | 10 |
| % Hispanic | 33 | 77 | 45 | 6 | 30 | 4 | 43 | 64 | 77 | 13 | 32 | 76 |
