# Supplementary material for: Neighborhood sampling: how many streets must an auditor walk?
Source: Int J Behav Nutr Phys Act. 2010 Mar 12;7:20. doi: 10.1186/1479-5868-7-20 (PMC3224902; doi:10.1186/1479-5868-7-20)
Supplement: Additional file 2 — Table S2. HD Neighborhood pedestrian built environment characteristics. Table S2 describes the pedestrian built environment characteristics of each housing development neighborhood. [file 1479-5868-7-20-S2.DOC]

^ segment contained many streets not publicly accessible (e.g., private business parkways), artificially inflating the node count.

| *Table 2*. HD Neighborhood pedestrian built environment characteristics | | | | | | | | | | | | |
| --- | --- | --- | --- | --- | --- | --- | --- | --- | --- | --- | --- | --- |
|  |  | **HD1** | **HD2** | **HD3** | **HD4** | **HD5** | **HD6** | **HD7** | **HD8** | **HD9** | **HD10** | **HD11** |
| **Characteristic** |  |  |  |  |  |  |  |  |  |  |  |  |
| Number of segments |  | 301 | 160 | 239 | 390 | 102 | 50 | 122 | 161 | 193 | 292 | 80 |
| Street node density |  | 329 | 204 | 269 | 529 | 237 | 576^ | 210 | 216 | 260 | 368 | 226 |
| % segments with sidewalks |  | 62 | 78 | 86 | 93 | 41 | 72 | 72 | 38 | 99 | 73 | 48 |
| % agree attractive for walking |  | 47 | 29 | 33 | 56 | 39 | 90 | 62 | 30 | 78 | 26 | 52 |
| % agree safe for walking |  | 56 | 48 | 35 | 65 | 36 | 62 | 65 | 50 | 89 | 53 | 81 |
| Mean connectivity (SD) |  | 1.6  (0.8) | 2.0  (1.0) | 1.2  (1.0) | 1.7  (0.9) | 0.4  (0.7) | 1.3  (0.7) | 1.7  (0.8) | 1.8  (0.9) | 2.6  (0.6) | 1.4  (0.8) | 0.6  (0.8) |
| Mean # lanes  (SD) |  | 2.1  (0.3) | 2.4  (0.6) | 2.2  (0.5) | 1.8  (0.6) | 2.2  (0.4) | 2.7  (0.5) | 2.2  (0.5) | 2.1  (0.3) | 2.3  (0.5) | 2.2  (0.4) | 2.1  (0.4) |
