# Supplementary material for: Neighborhood sampling: how many streets must an auditor walk?
Source: Int J Behav Nutr Phys Act. 2010 Mar 12;7:20. doi: 10.1186/1479-5868-7-20 (PMC3224902; doi:10.1186/1479-5868-7-20)
Supplement: Additional file 3 — Table S3. All HD and HD Neighborhood core vs. ring comparison of pedestrian built environment characteristics. Table S3 describes the core vs. ring comparisons of pedestrian built environment characteristics of all housing development neighborhoods and of each housing development neighborhood. [file 1479-5868-7-20-S3.DOC]

| *Table 3*. All HD and HD Neighborhood core vs. ring comparison of pedestrian built environment characteristics | | | | | | | | | | | | |
| --- | --- | --- | --- | --- | --- | --- | --- | --- | --- | --- | --- | --- |
| **Characteristic** | **All HD** | **HD1** | **HD2** | **HD3** | **HD4** | **HD5** | **HD6** | **HD7** | **HD8** | **HD9** | **HD10** | **HD11** |
| sidewalk presence (p value) | 0.0543* | 0.130 | 0.277 | 1.000 | 0.0115** | 0.235 | 0.723 | 0.091 | 0.700 | 0.192 | 0.0008** | 0.789 |
| Attractive for Walking in Core (% strongly agree or agree) | 43 | 61 | 31 | 38 | 43 | 43 | 100 | 56 | 20 | 75 | 10 | 40 |
| Attractive for Walking in Ring (% strongly agree or agree) | 47 | 41 | 28 | 31 | 65 | 36 | 84 | 66 | 34 | 79 | 35 | 56 |
| Attractive for walking | **⇓** | **⇑**** | **⇑** | **⇑** | **⇓**** | **⇑** | **⇑#** | **⇓** | **⇓** | **⇓** | **⇓**** | **⇓** |
| Safe for Walking in Core (% strongly agree or agree) | 51 | 64 | 49 | 30 | 50 | 46 | 67 | 49 | 35 | 84 | 40 | 80 |
| Safe for Walking in Ring (% strongly agree or agree) | 60 | 51 | 47 | 37 | 75 | 30 | 59 | 75 | 56 | 92 | 60 | 81 |
| safe for walking | **⇓**** | **⇑** | **⇑** | **⇓** | **⇓**** | **⇑** | **⇑** | **⇓**** | **⇓*** | **⇓** | **⇓**** | **⇓#** |
| connectivity (p value) | 0.224 | 0.059 | 0.0004** | 0.796 | 0.0055** | 0.212 | 0.060 | 0.0533* | 0.252 | 0.383 | 0.0051** | 0.708 |
| lanes (p value) | <.0001** | 0.059 | 0.0198* | 0.645 | <.0001** | 0.056 | 0.504 | 0.0053** | 0.207 | 0.278 | 0.547 | 0.144 |

⇑ core rated as more attractive or safe than ring (% strongly agree/agree for core greater than for ring)

⇓ core rated as less attractive or safe than ring (% strongly agree/agree for ring greater than for core)

* p<.05; ** p<.01

# contains cell size <5
