# Supplementary material for: Neighborhood sampling: how many streets must an auditor walk?
Source: Int J Behav Nutr Phys Act. 2010 Mar 12;7:20. doi: 10.1186/1479-5868-7-20 (PMC3224902; doi:10.1186/1479-5868-7-20)
Supplement: Additional file 4 — Table S4. Comparisons of core vs. random sample of core residential segments (75%, 50% and 25%). Table S4 describes the comparisons of residential street segments in the core and a random sample of 75%, 50%, 25% residential segments in the core, respectively. [file 1479-5868-7-20-S4.DOC]

| *Table 4*. Comparisons of core vs. random sample of core residential segments (75%, 50% and 25%) | | | | | | | | | | | | | |
| --- | --- | --- | --- | --- | --- | --- | --- | --- | --- | --- | --- | --- | --- |
| **Core vs. 75%** |  | **All HD** | **HD1** | **HD2** | **HD3** | **HD4** | **HD5** | **HD6** | **HD7** | **HD8** | **HD9** | **HD10** | **HD11** |
| sidewalk presence (p value) |  | 0.843 | 0.943 | 1.00# | 1.00# | 0.926 | 1.00 | 0.988# | 1.00# | 1.00 | 1.00# | 0.992 | 0.78# |
| Attractive for walking in the Core/75% (% strongly agree or agree) |  | **44/44** | **59/61** | **35/31** | **37/38** | **44/43** | **50/43** | **100/100** | **56/56** | **17/20** | **74/75** | **11/10** | **60/57** |
| attractive for walking |  | ⇔ | ⇓ | ⇑ | ⇓ | ⇑ | ⇑ | ⇔ | ⇔ | ⇓ | ⇓ | ⇑ | ⇑ |
| Safe for walking in the Core/75% (% strongly agree or agree) |  | **51/50** | **66/64** | **50/49** | **30/30** | **49/50** | **54/46** | **69/67** | **50/49** | **31/35** | **83/82** | **33/35** | **80/79** |
| safe for walking |  | ⇑ | ⇑ | ⇑ | ⇔ | ⇓ | ⇑ | ⇑ | ⇑ | ⇓ | ⇑ | ⇓ | ⇑ |
| connectivity (p value) |  | 0.878 | 0.995# | 0.867# | 0.691 | 0.966 | 0.987# | 0.90# | 0.957# | 0.698# | 0.973# | 0.972 | 0.553# |
| lanes (p value) |  | 0.84 | 1.00# | 0.908# | 0.608# | 0.661 | 0.702# | 1.00# | 0.763# | 1.00# | 0.523 | 0.755 | 1.00# |
| **Core vs. 50%** |  | **All HD** | **HD1** | **HD2** | **HD3** | **HD4** | **HD5** | **HD6** | **HD7** | **HD8** | **HD9** | **HD10** | **HD11** |
| sidewalk presence (p value) |  | 0.423 | 0.765 | 1.00 | 0.722# | 0.608 | 0.299 | ---- | 0.947 | 0.797 | 0.856# | 0.936 | 0.555# |
| Attractive for walking in the Core/50% (% strongly agree or agree) |  | **42/42** | **53/61** | **33/31** | **42/38** | **38/43** | **37/43** | **----** | **61/56** | **22/20** | **79/75** | **13/10** | **75/57** |
| attractive for walking |  | ⇔ | ⇓ | ⇑ | ⇑ | ⇓ | ⇓ | **----** | ⇑ | ⇑ | ⇑ | ⇑ | ⇑ |
| Safe for walking in the Core/50% (% strongly agree or agree) |  | **50/51** | **62/64** | **39/49** | **35/30** | **47/50** | **47/46** | **----** | **48/49** | **39/35** | **86/82** | **43/35** | **75/79** |
| safe for walking |  | ⇔ | ⇓ | ⇓ | ⇑ | ⇓ | ⇑ | **----** | ⇓ | ⇑ | ⇑ | ⇑ | ⇓ |
| connectivity (p value) |  | 0.681 | 0.94# | 0.966# | 0.964 | 0.868 | 0.908# | ---- | 0.46# | 0.931# | 0.836# | 0.477# | 0.73# |
| lanes (p value) |  | 0.71 | 0.235# | 0.593 | 0.78# | 0.725# | 0.515# | ---- | 0.74# | 0.378# | 0.496 | 0.642# | 0.644# |
| **Core vs. 25%** |  | **All HD** | **HD1** | **HD2** | **HD3** | **HD4** | **HD5** | **HD6** | **HD7** | **HD8** | **HD9** | **HD10** | **HD11** |
| sidewalk presence (p value) |  | 0.736 | 0.972 | ---- | 0.686# | 0.811 | 0.836# | ---- | 1.00# | 0.214# | 1.00# | 0.89 | ---- |
| Attractive for walking in the Core/25% (% strongly agree or agree) |  | **41/42** | **67/61** | **----** | **33/38** | **39/43** | **50/43** | **----** | **55/56** | **8/20** | **79/75** | **12/10** | **----** |
| attractive for walking |  | ⇓ | ⇑ | **----** | ⇓ | ⇓ | ⇑ | **----** | ⇓ | ⇓ | ⇑ | ⇑ | **----** |
| Safe for walking in the Core/25% (% strongly agree or agree) |  | **45/49** | **67/64** | **----** | **27/30** | **44/50** | **50/46** | **----** | **45/49** | **33/35** | **79/82** | **24/35** | **----** |
| safe for walking |  | ⇓ | ⇑ | **----** | ⇓ | ⇓ | ⇑ | **----** | ⇓ | ⇓ | ⇓ | ⇓ | **----** |
| connectivity (p value) |  |  |  |  |  |  |  |  |  |  |  |  |  |
| lanes (p value) |  |  |  |  |  |  |  |  |  |  |  |  |  |

⇑ core rated as more attractive or safe than smaller %age (% strongly agree/agree for core greater than for smaller %age)

⇓ core rated as less attractive or safe than smaller %age (% strongly agree/agree for smaller %age greater than for core)

⇔ core and smaller %age are equivalent in terms of attractiveness and feelings of safety

all values represented are p values; p<.05; ** p<.01

# contains cell size <5

Connectivity and lanes p values are not reported for the core vs. 25%, 50% and 75% samples because there were a large number of HDs with cell sizes <5, reducing the validity of the findings
